# Targeting the SET and RING-associated (SRA) domain of ubiquitin-like, PHD and ring finger–containing 1 (UHRF1) for anti-cancer drug development

**DOI:** 10.18632/oncotarget.25425

**Published:** 2018-05-25

**Authors:** Debasis Patnaik, Pierre-Olivier Estève, Sriharsa Pradhan

**Affiliations:** ^1^ Meliorate Inc., North Weymouth, MA, 02191, USA; ^2^ New England Biolabs Inc., Ipswich, MA, 01938, USA

**Keywords:** UHRF1, SRA domain, cancer, DNA methylation, epigenetic target

## Abstract

Ubiquitin-like containing PHD Ring Finger 1 (UHRF1) is a multi-domain protein with a methyl-DNA binding SRA (SET and RING-associated) domain, required for maintenance DNA methylation mediated by DNMT1. Primarily expressed in proliferating cells, UHRF1 is a cell-cycle regulated protein that is required for S phase entry. Furthermore, UHRF1 participates in transcriptional gene regulation by connecting DNA methylation to histone modifications. Upregulation of UHRF1 may serve as a biomarker for a variety of cancers; including breast, gastric, prostate, lung and colorectal carcinoma. To this end, overexpression of UHRF1 promotes cancer metastasis by triggering aberrant patterns of DNA methylation, and subsequently, silencing tumor suppressor genes. Various small molecule effectors of UHRF1 have been reported in the literature, although the mechanism of action may not be fully characterized. Small molecules that potentially bind to the SRA domain may affect the ability of UHRF1 to bind hemimethylated DNA; thereby reducing aberrant DNA methylation. Therefore, in a subset of cancers, small molecule UHRF1 inhibitors may restore normal gene expression and serve as useful anti-cancer therapeutics.

## INTRODUCTION

**Figure 1:**
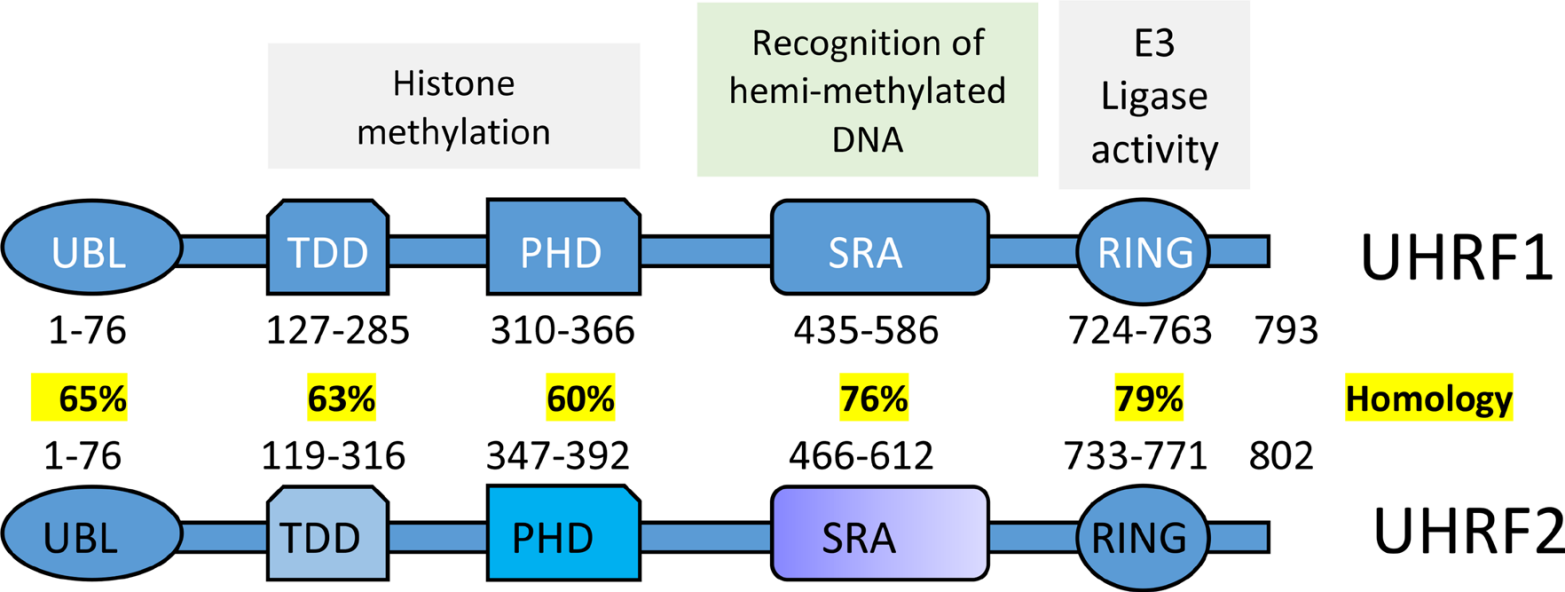
Schematics is showing multiple domains of UHRF1 and UHRF2 (Modified after Zhang *et al.* 2011 [11] (UBL, TTD, PHD, SRA, and RING are the abbreviations for the Ubiquitin-like domain, Tandem Tudor Domain, Plant Homeo Domain, SET and Ring Associated, and Really Interesting New Gene domain respectively). Interaction with DNMT1 leads UHRF1 but not UHRF2 to play a significant role in the maintenance of DNA methylation.

In recent years, multiple epigenetic effectors with potential pro-oncogenic functions have been targeted for anti-cancer drug development. These epigenetic effectors consist of writer, reader and eraser proteins that either generate, recognize or revert select histone or DNA modifications [1, 2]. One class of so-called epigenetic “writers” are the family of mammalian cytosine-5 DNA methyltransferases: DNMT1, 3a, and 3b. In this family, DNMT1 functions as the canonical maintenance methyltransferase and post-replicatively generates 5-methylcytosine (5mC) on newly synthesized daughter strands [3]. DNA methylation functions as a gene silencing mark, and aberrant global hypo- or hyper- cytosine methylation is a common hallmark of cancer. DNA methylation patterns influence post-translational modification of histones, gene expression, and chromatin compaction. UHRF1, also known as Np95 (Nuclear Protein 95), in mouse [4, 5] or ICBP90 (Inverted CCAAT box-binding protein of 90 kDa) in human [6–9] is essential for cell proliferation and DNA methylation maintenance. UHRF1 directly interacts with DNMT1 through its distinct structural domains; including a ubiquitin-like (UBL) domain, a plant homeodomain (PHD) domain, a SRA (SET and RING-associated) domain and a RING domain [10–14] (Figure 1). This interaction allows UHRF1 to recruit DNMT1 to hemimethylated DNA, and thusly, facilitates maintenance DNA methylation [12]. Furthermore, UHRF1 is one of the few proteins that recognize both histone and DNA modification marks on chromatin and can mediate epigenetic cross-talk [10, 11, 15–22]. UHRF1 depletion inhibits chromosomal DNA replication in Xenopus egg extracts [23] and plays a key role in transferring methylation status from mother cells to daughter cells. Genetic deletion of UHRF1 leads to a severe loss (~80%) of DNA methylation, despite the presence of functional DNA methyltransferases. Collectively, the molecular interplay between UHRF1 and DNMT1 is essential for reliable propagation of DNA methylation patterns, and furthermore, this direct interaction justifies the significance of UHRF1 as a therapeutic target [16, 24].

## ROLE OF UHRF1 IN CANCERS OR TUMORIGENESIS

**Figure 2:**
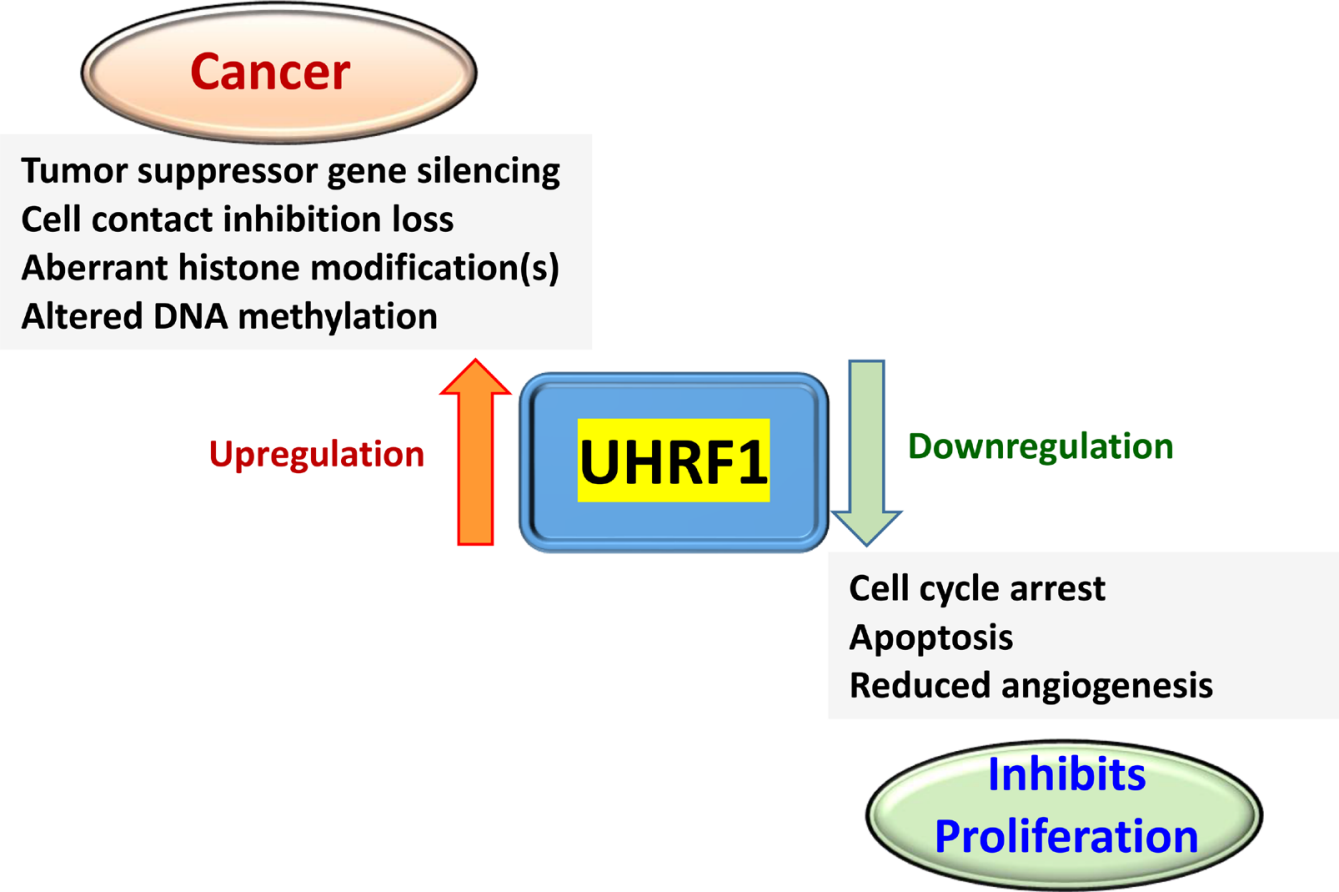
A simplified version of the events relevant for anti-cancer drug development associated with UHRF1 up- or downregulation

UHRF1 is an epigenomic regulator involved in multiple cellular processes that leads to tumor development (Figure 2). Imbalance of UHRF1 levels in cells plays a significant role in cancer initiation, metastasis, and tumor relapse [25]. In normal cells, UHRF1 is a cell-cycle regulated protein required for S-phase entry, which is primarily expressed during cell proliferation [26], and notably absent in G0 and G1 phases [4, 5, 27]. However, during tumorigenesis UHRF1 promotes proliferation of cancer cells and is abundantly expressed throughout cell cycle. Immunohistochemistry and microarray analysis of various tissues from cancer patients also supports UHRF1 overexpression in several cancer types, such as lung [16, 28, 29], breast [30], gastric [31], prostate [32, 33] and colorectal carcinomas [34]. To this effect, UHRF1 was suggested as a diagnostic biomarker for cervical [35], pancreatic [36], bladder [37] and lung cancers [29]. Therefore, the development of reliable, sensitive and non-invasive methods to detect UHRF1 may facilitate cancer diagnosis and disease prognosis. The pro-oncogenic role of UHRF1 is causally related to its role in establishment of DNA methylation; indeed, overexpression of UHRF1 facilitates coordinated tumor suppressor gene silencing in multiple cancers by altering DNA methylation patterns. In contrast, down-regulation of UHRF1 results in cell growth inhibition [38]. UHRF1-mediated silencing of tumor suppressor genes during cell division functions through recruitment of several repressor enzymes; such as histone deacetylase 1 (HDAC1), DNA methyltransferase 1 (DNMT1) and histone lysine methyltransferases, i.e., G9a and Suv39H1 [39]. Therefore, UHRF1 has attracted considerable attention as a potential anti-cancer drug target [40] and universal cancer biomarker. In the following examples, we will briefly discuss the role of UHRF1 in various types of cancer.

In breast cancer, UHRF1 has been identified as a bonafide biomarker [41]. Data derived from cDNA-microarray studies confirmed UHRF1 (ICBP90) overexpression in a variety of primary breast cancer samples [9]. Indeed, a large percentage of cells, analyzed from low and high grade breast carcinomas, highly express UHRF1, and additionally, UHRF1 expression levels matched the grade of cancer [8]. Elevated levels of UHRF1 DNA in plasma directly correlated with short progression-free survival of breast cancer patients [42]. Ultimately, breast cancer patients with high UHRF1 expression are likely to have a poor prognosis. Therefore, the level of UHRF1 DNA in plasma is substantially indicative of the status and stage of breast cancer; and may serve as a useful diagnostic and clinically prognostic marker of breast cancer [42].

In esophageal squamous cell carcinoma (ESCC), resistance to radiation therapy is directly associated with overexpression of UHRF1. Inhibition of UHRF1 expression via lentivirus-mediated shRNA significantly enhances radiosensitivity via alteration of cell cycle progression, higher rates of apoptosis, and a decreased capacity to repair DNA damage [43]. In ESCC cell lines, Nakamura and coworkers reported that vector-mediated overexpression of UHRF1 triggers global DNA hypomethylation, particularly at LINE-1 (long interspersed nucleotide element-1) elements [44]. The results are significant, as the methylation level of LINE-1 is regarded as a proxy marker for total DNA methylation and are being investigated as a prognostic biomarker for cancer. Most significantly in a cohort of ESCC patients, UHRF1 overexpression was correlated with poor prognosis [44, 45]. Thus, UHRF1 is an independent prognostic marker for ESCC and may be considered further as a possible therapeutic target in patients with higher levels of UHRF1 expression [45].

In gastric cancer (GC), overexpression of UHRF1 was reported in metastatic tissue [31], while downregulation of UHRF1 suppressed gastric cancer invasion and metastasis. Results from both *in vitro* and *in vivo* studies, confirmed that UHRF1 downregulation could suppress the development of gastric cancer [31]. More significantly, the level of UHRF1 overexpression corresponded directly with the stage of gastric cancer, being highest in stage IV and grade III. UHRF1 DNA levels measured in the serum of gastric cancer patients were substantially higher than those of healthy controls, and this finding is consistent with previous studies. These studies raise the possibility of monitoring UHRF1 expression, in at-risk patient serum and tissues, as a novel diagnostic and prognostic biomarker for gastric cancer [46].

In hepatocellular carcinoma (HCC), overexpression of UHRF1 facilitates DNA hypomethylation [47]. Knockdown of UHRF1, both *in vitro* and *in vivo*, inhibited cancer progression in hepatocellular carcinoma by inducing G2/M arrest during cell cycle. Furthermore, transcriptional upregulation and increased protein levels of UHRF1 seems to contribute towards a poorer patient prognosis by promoting cell proliferation and metastasis in HCC [48]. On a different note, downregulation of UHRF1 have also been reported to enhance the migratory and invasive properties of hepatocellular carcinoma and contributes to the development of cancer stem-like cells [49]. UHRF1 thus can be a potential prognostic biomarker for hepatocellular carcinoma [50].

Laryngeal squamous cell carcinoma (LSCC) is one of the most widespread cancers of the head and neck. Elevated transcription of UHRF1 mRNA was correlated with an advanced cancer stage, poor histological differentiation, and poor prognosis [51]. Therefore, UHRF1 may have a significant role in the progression of LSCC and may be utilized as a prognostic biomarker [51]. Integrated bioinformatics studies of 959 differentially-expressed genes (DEG) in non-cancerous vs. LSCC samples identified UHRF1, among one of five genes, with a probable causal association with the disease [52].

Similarly in leukemia, UHRF1-mediated DNA methylation has been shown to regulate self-renewal vs. differentiation cell fates of individual hematopoietic stem cells (HSC) via epigenetic regulation of HSC division [53]. Indeed, disruption of the balance between HSC self-renewal and differentiation is a definitive hallmark of oncogenesis [54]. Thus, UHRF1 may play a role in maintaining leukemic stem cells (LSCs) self-renewal via regulation of DNA methylation and may be considered as a potential target for leukemia treatment [53].

UHRF1 overexpression is characteristically observed in many forms of cancer, including lung carcinomas starting from an early pathological stage. Therefore, the detection of UHRF1 levels in tissue specimens could offer a unique diagnostic capability [29]. Indeed, lung carcinomas with elevated levels of UHRF1 develop the capacity to bypass cell contact inhibition; a typical feature observed in human lung fibroblasts that is associated with hypermethylation of cyclin-dependent kinase Inhibitor 2A (CDKN2A) and Ras association domain-containing protein 1 (RASSF1) promoters. UHRF1 is part of a six-gene signature for predicting a risk score that classifies non-small cell lung cancer (NSCLC) patients into either high or low-risk categories [55]. Microarray analysis of epithelial ovarian cancer (EOC) along with matched healthy controls has recently identified UHRF1 as one of the key differentially upregulated genes during disease pathogenesis [56]. The results highlighted that UHRF1 could act as a causative factor in ovarian cancer and as a suitable target for the development of anti-cancer therapies [57].

Proteomic analysis has identified UHRF1 as one of the top differentially expressed genes in chronic pancreatitis and pancreatic adenocarcinoma tissues, as compared to, normal healthy pancreatic tissue [36]. The results suggest the possibility of employing UHRF1 expression analysis, tissue samples acquired from the patients during endoscopy, as a diagnostic tool [36]. Cui and coworkers [58] reported elevated levels of UHRF1 expression in pancreatic cancer tissue samples, relative to adjacent healthy tissue samples. Moreover, UHRF1 expression in pancreatic cancer was inversely associated with patient survival. Results from both *in vitro* as well as *in vivo* studies have shown that UHRF1 supports pancreatic cancer metastasis and plays a causative role in the development of pancreatic carcinoma [58]. These studies provide a valid reason to consider targeting UHRF1 for the treatment of pancreatic cancer, lung cancer, and epithelial ovarian cancer.

Positive expression of UHRF1 has been detected immunohistochemically in a significant portion of tumors amongst 223 prostate tumor samples. Knockdown of UHRF1 in prostate cancer cells reduced their malignant characteristics, signifying its important role in prostate cancer progression [33]. Expression of UHRF1 correlates negatively with multiple tumor suppressors and positively with EZH2 expression (histone methyltransferase) in tumors and prostate cancer cell lines. Thus, UHRF1 along with the SUV39H1 histone methyltransferase, and DNA methyltransferases are thought to be involved in the epigenetic silencing of genes in prostate cancer [33].

UHRF1 is known to be highly expressed in retinoblastoma [59, 60], an intraocular tumor that arises from developing retina via inactivation of the RB1 gene. When compared with the methylome of normal retina, genomes of human primary retinoblastoma and cell lines have varied DNA methylation patterns characterized by global hypomethylation, but higher levels of methylation at the promoters of tumor suppressors genes [61]. All the above examples demonstrate the role of UHRF1 in oncogenesis. This multidomain protein plays a vital role in the epigenetic regulation of a variety of proteins leading to alteration of critical cell cycle pathways.

### Multidomain structure of UHRF1 offers unique opportunities and challenges for drug development

UHRF1 is a multidomain protein. The multifunctional cellular role of UHRF1 is attributed to its distinct structural domains that include a ubiquitin-like domain (also known as novel Np95/ICBP90-like RING finger protein N-terminus or NIRF_N), a tandem tudor domain (TTD), a plant homeodomain (PHD) domain, a SRA domain (SET [derived from Su(var)3-9, Enhancer-of-zeste and Trithorax], a RING-associated domain), and a RING (Really Interesting New Gene) domain [13] (Figure 1). The ubiquitin-like domain has received comparatively lesser attention than the other domains due to its conserved structure with ubiquitin and is thought to be involved in protein-protein interactions or participates in proteasomal protein turnover [13]. The plant homeodomain (PHD) domain of UHRF1 reportedly plays a significant role in reading the histone code by specifically binding methylated lysines [21, 62] and arginines [63]. The linked tandem tudor domain and plant homeodomain of UHRF1 were reported to act as a tandem functional unit in cells. Together, both domains provide a defined multivalent and combinatorial readout of a heterochromatin signature within a single histone H3 tail, and this functional cooperation is necessary for the UHRF1-directed epigenetic inheritance of DNA methylation [21]. UHRF1's TTD and PHD domains are required for specific recognition of trimethylated lysine within the N-terminus of histone H3 [10], and this may play a role in establishing and preserving histone H3K9 methylation patterns during the cell cycle [64]. Furthermore, UHRF1 exists in distinct active states, binding either unmodified H3 or the H3 lysine 9 trimethylation (H3K9me3) modification. A polybasic region (PBR) in the C-terminus blocks the interaction of the TTD domain with H3K9me3 by occupying an essential peptide-binding groove. In this state, the PHD domain mediates interaction with the extreme N-terminus of the unmodified H3 tail. Therefore, UHRF1 contains different H3K9 binding domains depending on the modification state of the histone itself. Hemimethylated DNA is reported to weaken the intramolecular molecular interaction of UHRF1, facilitating the opening of a closed conformation of UHRF1 that allows recognition of histone methylation marks by the TTD-PHD module [65]. The multiple domains of UHRF1 thus appear to act in a combinatorial fashion. The multivalent action of UHRF1 is regulated by interactions between various chromatin factors in different physiological settings, and in response to external signals [66].

UHRF1 is a member of the subfamily of RING (Really Interesting New Gene) finger-type E3 ubiquitin ligases. Ubiquitin ligases play a key role in the regulation of many cellular processes, including cell cycle progression, and are being investigated to discover new prognostic biomarker, and also to identify the next generation of therapeutic opportunities for the treatment of cancer [67]. The E3 ubiquitin ligase activity of the RING domain has been investigated as the preferred drug target owing to its detectable enzymatic activity and for its role in tumor growth [68]. The E3 ligase activity of the ring domain of UHRF1 promotes ubiquitination-mediated degradation of the promyelocytic leukemia (PML) protein, a tumor-suppressor protein implicated in tumorigenesis in multiple forms of cancer [69]. In *Xenopus* egg extracts, Uhrf1-dependent histone H3 ubiquitination has been reported to have a prerequisite role in the maintenance of DNA methylation [20]. DNA damage is reported to enhance phosphorylation of UHRF1, at serine 108, by casein kinase 1 delta (CK1δ), accelerating UHRF1 degradation. SCF^β-TrCP^ has been reported to be UHRF1 E3 ligase necessary for regulating UHRF1 steady-state levels under regular conditions and in response to DNA damage [25]. The molecular mechanism by which the level of cellular UHRF1 is regulated determines cell proliferation capability [70]. Recently Poly(ADP-ribose) Polymerase 1 (PARP1) was found to associate with UHRF1, and thereby, modulates UHRF1-regulated events associated with heterochromatin, namely by the accumulation of H4K20me3 and subsequent DNMT1 clearance [71]. A recent report [72] demonstrated that access to multiple histone H3-binding domains of UHRF1 is regulated allosterically by phosphatidylinositol 5-phosphate and that hemimethylated DNA plays a significant role in guiding inheritance of DNA methylation via allosteric activation of H3 ubiquitylation mediated by UHRF1.

UHRF1 shares similar domain structure with its paralog UHRF2 (Figure 1). However, UHRF2 has a unique role in the maintenance of 5-hydroxymethylcytosine (5hmC) levels that is distinct from its paralog UHRF1 [73]. A recent study provides evidence for the role of UHRF2 as a bonafide 5hmC reader [74]. UHRF2 is abundantly expressed in brain, and poorly expressed in several human cancers [75]. Structural study of the SRA domain of UHRF2 in complex with a 5hmC containing DNA, reveals a preferential binding facilitated by the formation of an optimal 5hmC binding pocket [76].

### SRA domain of UHRF1 as a drug target to prevent aberrant DNA methylation

The SET and RING-associated (SRA) domain of UHRF1 is a DNA-binding domain that has the capability to recognize 5-methylcytosine (5mC) in hemimethylated CpG dinucleotides [15, 77–80]. Hemimethylated DNA sequences are fully methylated by maintenance DNA methylation, where UHRF1 plays a significant role. UHRF1 preferentially binds to hemimethylated DNA compared to unmethylated and methylated DNA. Molecular dynamics simulations indicate that UHRF1 discriminates against binding to methylated CpG-Sites via steric repulsion [81]. The SRA domain detects the existence of a methylated cytosine on a single DNA strand and recruits DNMT1 which methylate the cytosine on the newly synthesized DNA [15, 78–80, 82]. During late S-phase, the binding specificity of the SRA domain allows UHRF1 to target DNMT1 to replication foci in a cell-cycle dependent manner [11]. Indeed, the SRA domain of UHRF1 has been shown to interact directly with DNMT1, which enables hemimethylated DNA to access the catalytic center of DNMT1 and stimulates DNA methyltransferase activity [83]. Interaction between DNMT1's replication foci targeting sequence (RFTS) with UHRF1's SRA domain, plays a significant role in the accurate feeding of the hemimethylated DNA to the catalytic center of DNMT1 [83]. *In vitro* studies reveal that UHRF1 can cause a 5-fold increase in the activity of DNMT1 and even the SRA domain alone can cause a 1.9-fold increase in DNMT1 activity. Moreover, the interaction between UHRF1 and DNMT1 leads to an approximately two-fold increase in the preference of DNMT1 for targeting hemimethylated DNA [84]. By this mechanism, UHRF1 has been shown to increase the activity and specificity of DNMT1 [78]. Therefore, preventing the interaction between the SRA domain and hemimethylated DNA, via small molecules, may prevent aberrant DNA methylation [84]. UHRF1 has been reported to bind H3K9me through mitosis to maintain 5mC [62]. UHRF1 can target DNMT1 for maintenance DNA methylation via binding either H3K9me2/3 and/or hemimethylated CpG and moreover, the presence of both binding activities safeguards the high fidelity maintenance of DNA methylation [22].

The SRA domain plays a key role in recognition and recruitment of UHRF1 to the DNA interstrand crosslinks (ICL) that result when cells are treated with DNA damaging reagents, such as mitomycin A and 4,5′,8-trimethylpsoralen (TMP) [85]. Results from a site-selective monitoring study using 2-aminopurine (2-Ap), a fluorescent analog of adenine, provided additional evidence for a reader role of the SRA domain in seeking hemimethylated CpG sites in the DNA sequences without any significant conformational and dynamical changes [86].

**Figure 3:**
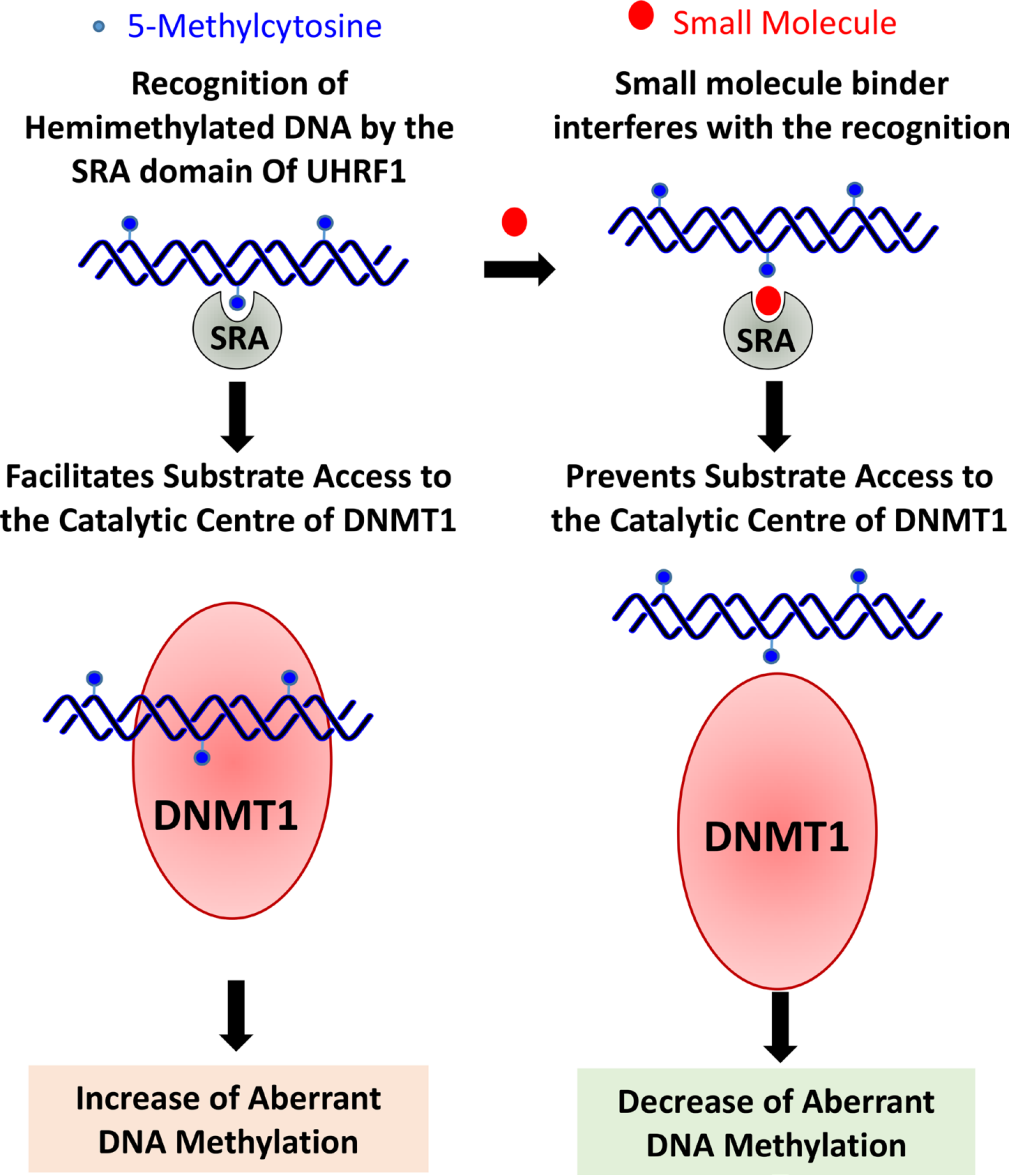
Small molecule binders of SRA domain of UHRF1 are expected to be effective in cancers where overexpression of UHRF1 leads to aberrant DNA methylation The small molecules may reduce aberrant methylation levels by preventing interaction of 5-methylcytosine with the SRA domain and thereby preventing substrate (hemimethylated DNA) access to the catalytic center of DNMT1. Moreover, these small molecule binders are likely to cause minimal disruption of epigenomic balance when compared with that may result due to direct inhibition of DNMT1, as levels of UHRF1 in a cell are quite less when compared with DNMT1 [40].

Multiple studies have highlighted the significant role of the SRA domain in regulating DNA methylation levels, and thus a small molecule binder of the SRA domain should be able to significantly reduce aberrant DNA methylation and emerge as a potential therapeutic target. A small molecule that can prevent the recognition of 5mC by the SRA domain could prevent binding of DNMT1 to the SRA domain of UHRF1, and therefore resulting in both reduced DNMT1 catalytic activity and reduced targeting towards hemimethylated DNA. More significantly, expression levels of *UHRF1* were reported to be 5–70 folds lower than those of *HDAC1* and *DNMT1* in all normal tissues. Therefore, any side effects that would arise via inhibiting UHRF1 expression or function are expected to be relatively tolerable [40]. UHRF1 is a promising target for anticancer therapy as discussed before. Due to 5mC binding epitope architecture, the SRA domain is a highly promising site for small molecules targeting [87]. The reversibility of epigenetic patterns offers a viable opportunity for therapeutic applications. Therefore, small molecule therapeutics, targeting UHRF1 and/or the SRA domain, could emerge as a low-risk anti-cancer therapy (Figure 3).

The potential of reversing DNA methylation and subsequently, reversing proportional gene offers an attractive option for clinical treatment of malignancies [88]. Combination strategies for anticancer drugs are a promising therapeutic approach, as it is likely to lower the concentration of each drug used and, in turn, reduce side effects [88]. FDA-approved drugs 5-azacytidine and decitabine (5-aza-2′-deoxycytidine) act as hypomethylating agents by inhibition of DNMT1 activity. Thus, a synergistic effect may be achieved by using a low dose of a DNMT1 inhibitor along with a small molecule candidate that can reduce aberrant methylation indirectly, by preventing the binding of DNMT1 to the SRA domain.

## SMALL MOLECULES EFFECTORS OF UHRF1

Natural products well known for exhibiting anti-cancer properties have been investigated for their ability to cause the downregulation of UHRF1 and re-expression of tumor suppressor gene [38]. Below, we summarize the small molecules effectors for UHRF1 (Table 1).

**Table 1:** List of known UHRF1 effectors

Name	Pubchem CID	Reference
Anisomycin	53602	[89]
Curcumin	969516	[90]
Dihydroartemisinin	456410	[91]
CD47, an integrin-associated protein		[92]
Emodin	3220	[93]
Factor quinolinone inhibitor 1	656346	[96]
Grape pomace extract		[97]
Hinokitiol (beta-Thujaplicin)	3611	[98]
*Limoniastrum guyonianum* aqueous gall extract		[99]
Naphthazarin	10141	[100]
Torin 2	51358113	[101]
4-benzylpiperidine-1-carboximidamide (BPC)		[104]
NSC232003 (5Z)-5-[1-(hydroxyamino)ethylidene]pyrimidine-2,4-dione)		[87]
Luteolin	5280445	[38]
Shikonin	479503	[102]

### Anisomycin

Anisomycin is an antibiotic produced by various *Streptomyces* sp. that acts by inhibition of protein synthesis and has the ability to activate signal transduction pathways (PubChem CID: 53602). Anisomycin inhibits proliferation of mammalian cells, via an unknown mechanism. Yu and coworkers [89] reported that anisomycin might be acting via activation of the P53/P21/P27 signaling pathway to decrease the expression of the ICBP90 (UHRF1) in Jurkat T cells. Expression of ICBP90 expression was found to decrease in a concentration-dependent manner following a 12 h treatment with anisomycin.

### Curcumin

Curcumin is a plant-derived polyphenol pigment isolated from the rhizome of turmeric (*Curcuma longa).* Curcumin has been shown to have multiple cellular targets and is one of the most powerful chemopreventive agents that inhibits the growth and proliferation of tumor cells (PubChem CID: 969516). Anti-proliferative effect of curcumin on melanoma cells has been documented by direct inhibition of PDE1, a regulator of UHRF1. Curcumin was found to decrease the expressions of PDE1A, cyclin A, UHRF1, and DNMT1 in melanoma cells. The observations were further supported by overexpression of PDE1, which leads to an increased level of UHRF1 expression resulting in reversing the anti-proliferative effects of curcumin [90].

### Dihydroartemisinin

Dihydroartemisinin is a derivative of artemisinin isolated from the plant *Artemisia annua*, a herb well-known in Chinese traditional medicine. Therapies using artemisinin-derived compounds are currently regarded as the worldwide standard for treating malaria caused by *Plasmodium falciparum* (PubChem CID: 456410). Du *et al.* [91] investigated the effects of an anticancer agent dihydroartemisinin towards inhibiting the expression of UHRF1 in human prostate cancer PC-3 cells. Dihydroartemisinin induced downregulation of UHRF1 and DNMT1, induced apoptosis, and G1/S cell-cycle arrest in PC-3 cells. These results suggested that downregulation of UHRF1/DNMT1 is upstream to many cellular events, including G1 cell arrest, demethylation of p16INK4A, and apoptosis. This study provided evidence that dihydroartemisinin can act as potential therapeutic agent in the treatment of prostate cancer, perhaps by influencing UHRF1 expression.

### Downregulation of UHRF1 via CD47, an integrin-associated protein

UHRF1 plays a crucial role in the silencing of multiple tumor suppressor genes including p16(INK4A), thus promoting cell proliferation. CD47 is an integrin-associated protein that is frequently over-expressed in various tumors including glioblastomas. In human astrocytoma cell lines, activation of CD47 increases the expression of UHRF1, thus correlating with the down-regulation of p16(INK4A) [92]. Antibody-based blocking of CD47 led to down-regulation of UHRF1 expression, concurrent with a re-expression of p16(INK4A), leading to decreased cell proliferation in both cancer cell lines. Interestingly, CD47 activated the inflammatory genes (IL-6, IL-7, and MCP-1) via an NF-kappaB dependent mechanism in human astrocytoma cancer cells but did not activate the same pathway in healthy cells. More significantly, neither CD47 activation nor its blocking had any effect on expression of UHRF1/p16(INK4A) in normal human astrocytes. The results highlight the potential of UHRF1 downregulation as a feasible therapeutic strategy.

### Emodin

Emodin is an anthraquinone found in several plants that was previously utilized as a laxative and currently employed as a tool in toxicity studies (PubChem CID: 3220). A recent study reported the role of emodin in promoting the arrest of human lymphoma Raji cell proliferation via the UHRF1-DNMT3A-ΔNp73 pathways [93]. Emodin was found to downregulate UHRF1, and therefore lead to a decrease in the lymphoma Raji cell viability, induction of apoptosis, and increased the activation of caspase-3, caspase-9 and poly (ADP-ribose) polymerase. Additionally, the emodin-induced downregulation of UHRF1 caused an increase of the DNMT3A levels, leading to an inhibition of the p73 promoter 2 activity, and decreased the levels of amino terminal truncated dominant-negative p73.

### Factor quinolinone inhibitor 1 (FQI1)

LSF (Late SV40 Factor), is a transcription factor and a novel oncogene for hepatocellular carcinoma [94]. Grant and coworkers [95] reported the identification of a lead compound designated as factor quinolinone inhibitor 1 (PubChem CID: 656346) that inhibited both the *in vitro* DNA-binding activity, as well as, the cellular activity of LSF. LSF has been experimentally shown to bind directly to both DNMT1 and UHRF1 both *in vivo* and *in vitro* [96]. Addition of FQI1 to the cell culture disrupted DNMT1 and UHRF1 complexes bound to LSF, leading to global aberrant CpG methylation and promoting altered cell cycle progression.

### Grape pomace extract

Grape pomace is formed during wine production process and mostly consists of the remaining part of the grape seeds, skins, and stems. Grape pomace has multiple bioactive ingredients, such as flavanols and anthocyanins, and thus was investigated for anti-cancer properties. In a recent study carried out in acute lymphoblastic leukemia Jurkat cells, Leon-Gonzalez *et al.* [97] reported that purified white grape pomace extract (PWGPE) reduced the expression of multiple proteins, thereby blocking the expression of crucial genes such as DNMT1, HDAC1/2, UHRF1, along with, members of the polycomb group (EZH2, SUZ12, and BMI1).

### Hinokitiol (beta-Thujaplicin)

Hinokitiol is a molecule typically found in fruits (PubChem CID: 3611). In colon cancer cells, hinokitiol treatment was reported to lower DNA methylation levels by inhibiting the expression of DNMT1 and UHRF1; and increasing the expression of ten-eleven translocation protein 1 (TET1). TET1 is involved in active DNA demethylation pathways by subsequent enzymatic oxidation of 5mC to 5-hydroxymethylcytosine (5hmC), 5-formylcytosine (5fC), and finally, 5-carboxycytosine (5caC) [98]. Evidence from ELISA and FACS data revealed that hinokitiol treatment did not cause any change in the levels of 5mC, but did increase the 5hmC level in the colon cancer cells.

### Limoniastrum guyonianum aqueous gall extract (G extract)

The anti-cancer effect of G extract and luteolin were evaluated in the human cervical cancer HeLa cell line. The results demonstrated inhibition of cell proliferation and induced G2/M cell cycle arrest in a concentration and time-dependent manner. Moreover, G extract- and luteolin-induced UHRF1 and DNMT1 downregulation occurred concurrently with global DNA hypomethylation in HeLa cells. These results were attributed to the growth inhibitory effects of G extract due to the activation of a p16INK4A -dependent cell cycle checkpoint signaling pathway caused by UHRF1 and DNMT1 down-regulation [99].

### Naphthazarin

When combined with ionizing radiation, naphthazarin (PubChem CID: 10141) induced cell cycle arrest and apoptosis in MCF-7 breast cancer cells. Results from ChIP assays demonstrated that a combined treatment of naphthazarin and ionizing radiation by inhibiting the binding of DNMT1, UHRF1, and HDAC1 to the p21 promoter [100], resulting in the increased expression of p21.

### Torin 2

Torin-2 is a novel mTOR (mammalian target of rapamycin) inhibitor (PubChem CID: 51358113). In addition to targeting the mTOR complex 1 (mTORC1), Torin-2 has been reported to induce autophagy and downregulate UHRF1 expression. An elevated level of UHRF1 expression is observed in hepatocellular carcinoma (HCC). Thus the down-regulation of UHRF1 may contribute towards the anticancer effect of Torin-2 [101], as evidenced by reduced proliferation and an increase in apoptosis.

### Shikonin

Shikonin, a plant-derived natural naphthoquinone (PubChem CID: 479503), is known to suppress the growth of multiple cancer types. Jang and coworkers [102] reported that shikonin causes apoptosis by up-regulating p73 and down-regulating UHRF1 in human cancer cells [102]. A recent report highlights the evaluation of shikonin derivatives as potential anti-neoplastic agents against various human cancer cell lines including breast, gastric and liver carcinoma [103].

### 4-benzylpiperidine-1-carboximidamide (BPC)

Using a fragment screening approach, Houliston *et al.* (2017) recently identified a compound, 4-benzylpiperidine-1-carboximidamide (BPC), which is capable of binding to the TTD groove of UHRF1 competing with linker binding, and thereby promoting open TTD-PHD conformations, which are comparatively less efficient at H3K9me3 binding [104]. Molecules capable of allosterically targeting H3K9me3 binding function of UHRF1 via the dynamic TTD-PHD domain module offer another avenue to modulate the histone reader function for therapeutic or experimental purposes.

### NSC232003

Myrianthopoulos and coworkers [87] used tandem virtual screening to identify *NSC232003,* a uracil derivative and a cell active molecule that binds to the 5mC binding pocket of the SRA domain of UHRF1 with high affinity. This is the first report of a small molecule that targets UHRF1 and modulates DNA methylation in a cellular context by possibly disrupting the interaction of DNMT1 with the SRA domain [87].

### Luteolin

Luteolin and flavonoids are well reported in the literature for the ability to downregulate UHRF1. The potential therapeutic benefits of targeting expression of UHRF1 via multiple natural products have been discussed earlier [38]. Flavonoids are plant-derived natural products that are known to influence various cellular targets. Luteolin is well documented in the literature as one of the leading polyphenolic effectors of UHRF1. In rat PC12 cells, luteolin treatment results in cell death, DNA fragmentation, and formation of apoptotic bodies [105]. Luteolin is also reported to inhibit cell proliferation, induce apoptosis and cell cycle arrest in G2/M phase via down-regulation of mitochondrial membrane potential in esophageal squamous cell carcinoma (ESCC) [106]. Moreover, luteolin inhibited the growth of ESCC tumors in xenograft mouse models without any systemic toxicity demonstrating its efficacy. Additionally, Luteolin was reported to inhibit proliferation of liver cancer cell lines SMMC-7721 and BEL-7402 in a time- and dose-dependent manner by arresting cell cycle at phase G1/S, enhancing Bax level, reducing anti-apoptotic protein Bcl-2 levels, activating caspase-3 enzymes and decreasing of mitochondrial membrane potential leading to cell apoptosis [107]. A comparative proteomics study recently reported that luteolin inhibited colorectal cancer cell epithelial-to-mesenchymal transition by suppressing CREB1 expression [108]. Luteolin inhibits the growth of the leukemic cell lines through induction of apoptosis most likely by inhibiting Ribosomal S6 kinases (RSK1) pathways and suppresses cell migration [109]. Luteolin and quercetin both inhibit the metastatic invasion of cervical cancer by reducing expression of ubiquitin E2S ligase (UBE2S) through epithelial-mesenchymal transition signaling [110]. Recently gastric cancer cell xenograft mouse model demonstrated the effectiveness of luteolin-induced inhibition of tumor growth *in vivo* [111]. Luteolin recently reported to inhibit lung metastasis and cell migration. Despite an abundance of articles on luteolin, the exact mechanism by which luteolin exerts anti-cancer activity is still unknown. Luteolin is likely to act as an anti-cancer agent by interaction with multiple cellular targets, and it is more than likely that luteolin may directly interact with UHRF1 by binding to the SRA domain. Luteolin prevents tumor development mostly by inactivating several signals and transcription pathways essential for cancer cells. Our studies indicate that luteolin may directly interact with the SRA domain, and thus contribute towards its anti-cancer activity.

## STRUCTURE-BASED STUDIES INDICATE LUTEOLIN AND TAXIFOLIN ARE LIKELY TO BIND THE SRA DOMAIN

**Figure 4:**
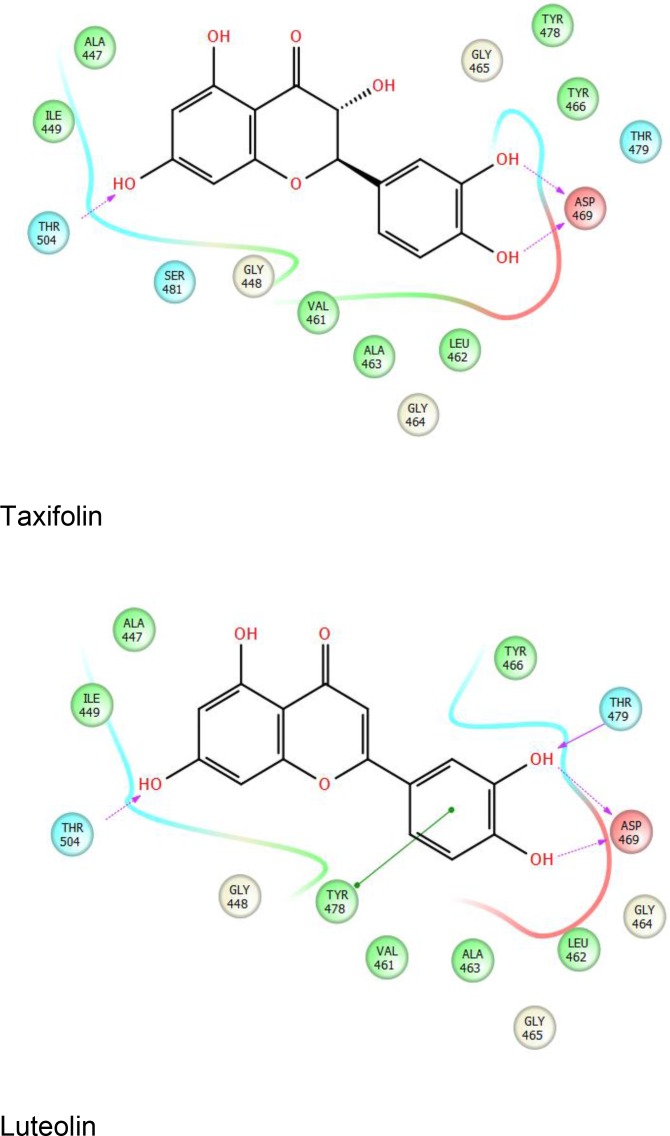
Representative ligand interaction diagrams with taxifolin (top) and luteolin (bottom) with the SRA domain of UHRF1

The structure of the SRA domain of UHRF1 and its interaction with hemimethylated DNA were the subjects of multiple concurrent investigations [15, 78, 79]. Our preliminary work identified luteolin and taxifolin among the top hits that are predicted to bind to the SRA domain of UHRF1 via structure-based screening of approximately 2.4 million compounds from multiple libraries from ChemDiv and TIMTEC, using Schrodinger's Small Molecule Drug Discovery Suite (Figure 4). The SDF format libraries of compounds were prepared with LigPrep, which generated accurate 3D molecular models for screening. Epik was used for reliable prediction of pKa values and to return all chemically sensible structures. Following the elimination of reactive molecules via filtering, remaining compounds were analyzed via QIKPROP analysis that predicted ADME (Absorption, Distribution, Metabolism, and Excretion) properties of the compounds. Virtual screening was performed using Schrodinger's virtual screening workflow that involves running Glide HTVS (High throughput virtual screening), Glide SP (standard precision) and Glide XP (Extra precision) successively on the compound database. This combinatorial screening workflow eliminated 90% of the compounds leaving only the top fraction (10%) of compounds on to subsequent stages. All the small molecule libraries were screened with the structure 3DWH [80] following processing via protein preparation wizard (Schrodinger) that ensured structural accuracy at the beginning of the screening project. The residue Asp469 that forms a hydrogen bond with methylcytosine [79] was defined as the active site, and an initial grid was prepared 10° from the selected residue (D469). The other residues of interest were Tyr466 and Tyr478 that sandwich 5mC; and Thr479, which is involved in preferential recognition of cytosine [79].

Results from an experimental colon carcinogenesis study support taxifolin as a chemopreventive agent via modulating inflammatory, Wnt and antioxidant response pathway proteins in the tumor microenvironment [112]. In pediatric Ewing's sarcoma (EWS) that affects soft tissues and bones, the combination of EWS oncogene knockdown and taxifolin treatment in cell cultures resulted in lower p53 promoter DNA methylation and triggered expression of apoptotic effectors, Puma and Noxa [113]. Moreover, in animal models, shRNA knockdown of EWS plus taxifolin treatment inhibited the growth of Ewing's sarcoma tumors due to an inhibition of angiogenic and invasive factors, and by inducing the activation of caspase-3 for apoptosis [113]. Results of this study by Hossain and Ray (2014) linked taxifolin with a decrease in DNA methylation. Flavonoids such as taxifolin are known to influence a variety of biological pathways, and thus the results from the above studies may also be due to a possible interaction of taxifolin with the SRA domain of UHRF1. Additional investigation will greatly help to confirm if the mode of action of taxifolin involves a direct interaction with the SRA domain. Nanoformulation-based technologies that improve the bioefficacy [114] are expected to play a greater role in the evaluation of natural products particularly the anti-cancer properties of taxifolin which are sometimes eclipsed in an experimental setting due to solubility, bioavailability and pharmacokinetic issues [115, 116].

## PROSPECTS

Aberrant DNA methylation is one of the foremost predictors of cancer development. In recent years, small molecule inhibitors of DNA methyltransferase have been approved by the FDA as drugs for the treatment of select cancers. However, treatment of pathological conditions caused by an aberrant pattern of DNA methylation is still in its infancy. DNMT1 inhibitors, such as decitabine, are subjects of clinical trials in several interventional studies either alone, or in combination with another drug or treatment. As per the clinicaltrials.gov website, DNA methylation biomarkers are the subjects of investigation for cervical, colorectal, gastric and stomach cancer. As a multifunctional and multidomain protein, UHRF1 plays a key role in connecting DNA methylation with multiple post-translational histone modifications. Overexpression of UHRF1 in cells leads to a reduction of maintenance DNA methylation levels and establishes an aberrant methylation pattern in the epigenome. Small molecules that bind to SRA domain are expected to prevent binding of hemimethylated DNA to UHRF1; thereby restoring normal DNA methylation levels. Furthermore, these molecules will aid in re-expression of previously silenced tumor suppressors. In summary, the SRA domain of UHRF1 is emerging as an attractive target to treat cancers characterized by UHRF1 overexpression and DNA hypermethylation. Small molecules binders of SRA domain of UHRF1 would be useful components of molecular toolbox for the study of cancer epigenetics, cell signaling pathways, and most significantly as lead molecules for the development of anti-cancer therapeutics.

## References

[1] Arrowsmith CH, Bountra C, Fish PV, Lee K, Schapira M (2012). Epigenetic protein families: a new frontier for drug discovery. Nat Rev Drug Discov.

[2] Zhang G, Pradhan S (2014). Mammalian epigenetic mechanisms. IUBMB Life.

[3] Pradhan S, Esteve PO (2003). Mammalian DNA (cytosine-5) methyltransferases and their expression. Clin Immunol.

[4] Fujimori A, Matsuda Y, Takemoto Y, Hashimoto Y, Kubo E, Araki R, Fukumura R, Mita K, Tatsumi K, Muto M (1998). Cloning and mapping of Np95 gene which encodes a novel nuclear protein associated with cell proliferation. Mamm Genome.

[5] Uemura T, Kubo E, Kanari Y, Ikemura T, Tatsumi K, Muto M (2000). Temporal and spatial localization of novel nuclear protein NP95 in mitotic and meiotic cells. Cell Struct Funct.

[6] Hopfner R, Mousli M, Garnier JM, Redon R, du Manoir S, Chatton B, Ghyselinck N, Oudet P, Bronner C (2001). Genomic structure and chromosomal mapping of the gene coding for ICBP90, a protein involved in the regulation of the topoisomerase IIalpha gene expression. Gene.

[7] Hopfner R, Mousli M, Oudet P, Bronner C (2002). Overexpression of ICBP90, a novel CCAAT-binding protein, overcomes cell contact inhibition by forcing topoisomerase II alpha expression. Anticancer Res.

[8] Mousli M, Hopfner R, Abbady AQ, Monte D, Jeanblanc M, Oudet P, Louis B, Bronner C (2003). ICBP90 belongs to a new family of proteins with an expression that is deregulated in cancer cells. Br J Cancer.

[9] Unoki M, Nishidate T, Nakamura Y (2004). ICBP90, an E2F-1 target, recruits HDAC1 and binds to methyl-CpG through its SRA domain. Oncogene.

[10] Cheng J, Yang Y, Fang J, Xiao J, Zhu T, Chen F, Wang P, Li Z, Yang H, Xu Y (2013). Structural insight into coordinated recognition of trimethylated histone H3 lysine 9 (H3K9me3) by the plant homeodomain (PHD) and tandem tudor domain (TTD) of UHRF1 (ubiquitin-like, containing PHD and RING finger domains, 1) protein. J Biol Chem.

[11] Zhang J, Gao Q, Li P, Liu X, Jia Y, Wu W, Li J, Dong S, Koseki H, Wong J (2011). S phase-dependent interaction with DNMT1 dictates the role of UHRF1 but not UHRF2 in DNA methylation maintenance. Cell Res.

[12] Bostick M, Kim JK, Esteve PO, Clark A, Pradhan S, Jacobsen SE (2007). UHRF1 plays a role in maintaining DNA methylation in mammalian cells. Science.

[13] Bronner C, Achour M, Arima Y, Chataigneau T, Saya H, Schini-Kerth VB (2007). The UHRF family: oncogenes that are drugable targets for cancer therapy in the near future?. Pharmacol Ther.

[14] Sharif J, Muto M, Takebayashi S, Suetake I, Iwamatsu A, Endo TA, Shinga J, Mizutani-Koseki Y, Toyoda T, Okamura K, Tajima S, Mitsuya K, Okano M (2007). The SRA protein Np95 mediates epigenetic inheritance by recruiting Dnmt1 to methylated DNA. Nature.

[15] Hashimoto H, Horton JR, Zhang X, Bostick M, Jacobsen SE, Cheng X (2008). The SRA domain of UHRF1 flips 5-methylcytosine out of the DNA helix. Nature.

[16] Unoki M, Brunet J, Mousli M (2009). Drug discovery targeting epigenetic codes: the great potential of UHRF1, which links DNA methylation and histone modifications, as a drug target in cancers and toxoplasmosis. Biochem Pharmacol.

[17] Rottach A, Frauer C, Pichler G, Bonapace IM, Spada F, Leonhardt H (2010). The multi-domain protein Np95 connects DNA methylation and histone modification. Nucleic Acids Res.

[18] Bronner C, Fuhrmann G, Chedin FL, Macaluso M, Dhe-Paganon S (2010). UHRF1 Links the Histone code and DNA Methylation to ensure Faithful Epigenetic Memory Inheritance. Genet Epigenet.

[19] Arita K, Isogai S, Oda T, Unoki M, Sugita K, Sekiyama N, Kuwata K, Hamamoto R, Tochio H, Sato M, Ariyoshi M, Shirakawa M (2012). Recognition of modification status on a histone H3 tail by linked histone reader modules of the epigenetic regulator UHRF1. Proc Natl Acad Sci U S A.

[20] Nishiyama A, Yamaguchi L, Sharif J, Johmura Y, Kawamura T, Nakanishi K, Shimamura S, Arita K, Kodama T, Ishikawa F, Koseki H, Nakanishi M (2013). Uhrf1-dependent H3K23 ubiquitylation couples maintenance DNA methylation and replication. Nature.

[21] Rothbart SB, Dickson BM, Ong MS, Krajewski K, Houliston S, Kireev DB, Arrowsmith CH, Strahl BD (2013). Multivalent histone engagement by the linked tandem Tudor and PHD domains of UHRF1 is required for the epigenetic inheritance of DNA methylation. Genes Dev.

[22] Liu X, Gao Q, Li P, Zhao Q, Zhang J, Li J, Koseki H, Wong J (2013). UHRF1 targets DNMT1 for DNA methylation through cooperative binding of hemi-methylated DNA and methylated H3K9. Nat Commun.

[23] Taylor EM, Bonsu NM, Price RJ, Lindsay HD (2013). Depletion of Uhrf1 inhibits chromosomal DNA replication in Xenopus egg extracts. Nucleic Acids Res.

[24] Sidhu H, Capalash N (2017). The key regulator of epigenetics and molecular target for cancer therapeutics. Tumour Biol.

[25] Chen H, Ma H, Inuzuka H, Diao J, Lan F, Shi YG, Wei W, Shi Y (2013). DNA damage regulates UHRF1 stability via the SCF(beta-TrCP) E3 ligase. Mol Cell Biol.

[26] Bonapace IM, Latella L, Papait R, Nicassio F, Sacco A, Muto M, Crescenzi M, Di Fiore PP (2002). Np95 is regulated by E1A during mitotic reactivation of terminally differentiated cells and is essential for S phase entry. J Cell Biol.

[27] Miura M, Watanabe H, Sasaki T, Tatsumi K, Muto M (2001). Dynamic changes in subnuclear NP95 location during the cell cycle and its spatial relationship with DNA replication foci. Exp Cell Res.

[28] Daskalos A, Oleksiewicz U, Filia A, Nikolaidis G, Xinarianos G, Gosney JR, Malliri A, Field JK, Liloglou T (2011). UHRF1-mediated tumor suppressor gene inactivation in nonsmall cell lung cancer. Cancer.

[29] Unoki M, Daigo Y, Koinuma J, Tsuchiya E, Hamamoto R, Nakamura Y (2010). UHRF1 is a novel diagnostic marker of lung cancer. Br J Cancer.

[30] Li XL, Xu JH, Nie JH, Fan SJ (2012). Exogenous expression of UHRF1 promotes proliferation and metastasis of breast cancer cells. Oncol Rep.

[31] Zhou L, Zhao X, Han Y, Lu Y, Shang Y, Liu C, Li T, Jin Z, Fan D, Wu K (2013). Regulation of UHRF1 by miR-146a/b modulates gastric cancer invasion and metastasis. FASEB J.

[32] Jazirehi AR, Arle D, Wenn PB (2012). UHRF1: a master regulator in prostate cancer. Epigenomics.

[33] Babbio F, Pistore C, Curti L, Castiglioni I, Kunderfranco P, Brino L, Oudet P, Seiler R, Thalman GN, Roggero E, Sarti M, Pinton S, Mello-Grand M (2012). The SRA protein UHRF1 promotes epigenetic crosstalks and is involved in prostate cancer progression. Oncogene.

[34] Kofunato Y, Kumamoto K, Saitou K, Hayase S, Okayama H, Miyamoto K, Sato Y, Katakura K, Nakamura I, Ohki S, Koyama Y, Unoki M, Takenoshita S (2012). UHRF1 expression is upregulated and associated with cellular proliferation in colorectal cancer. Oncol Rep.

[35] Li XL, Meng QH, Fan SJ (2009). Adenovirus-mediated expression of UHRF1 reduces the radiosensitivity of cervical cancer HeLa cells to gamma-irradiation. Acta Pharmacol Sin.

[36] Crnogorac-Jurcevic T, Gangeswaran R, Bhakta V, Capurso G, Lattimore S, Akada M, Sunamura M, Prime W, Campbell F, Brentnall TA, Costello E, Neoptolemos J, Lemoine NR (2005). Proteomic analysis of chronic pancreatitis and pancreatic adenocarcinoma. Gastroenterology.

[37] Yang GL, Zhang LH, Bo JJ, Chen HG, Cao M, Liu DM, Huang YR (2012). UHRF1 is associated with tumor recurrence in non-muscle-invasive bladder cancer. Med Oncol.

[38] Alhosin M, Sharif T, Mousli M, Etienne-Selloum N, Fuhrmann G, Schini-Kerth VB, Bronner C (2011). Down-regulation of UHRF1, associated with re-expression of tumor suppressor genes, is a common feature of natural compounds exhibiting anti-cancer properties. J Exp Clin Cancer Res.

[39] Alhosin M, Omran Z, Zamzami MA, Al-Malki AL, Choudhry H, Mousli M, Bronner C (2016). Signalling pathways in UHRF1-dependent regulation of tumor suppressor genes in cancer. J Exp Clin Cancer Res.

[40] Unoki M (2011). Current and potential anticancer drugs targeting members of the UHRF1 complex including epigenetic modifiers. Recent Pat Anticancer Drug Discov.

[41] Jin W, Chen L, Chen Y, Xu SG, Di GH, Yin WJ, Wu J, Shao ZM (2010). UHRF1 is associated with epigenetic silencing of BRCA1 in sporadic breast cancer. Breast Cancer Res Treat.

[42] Geng Y, Gao Y, Ju H, Yan F (2013). Diagnostic and prognostic value of plasma and tissue ubiquitin-like, containing PHD and RING finger domains 1 in breast cancer patients. Cancer Sci.

[43] Yang C, Wang Y, Zhang F, Sun G, Li C, Jing S, Liu Q, Cheng Y (2013). Inhibiting UHRF1 expression enhances radiosensitivity in human esophageal squamous cell carcinoma. Mol Biol Rep.

[44] Nakamura K, Baba Y, Kosumi K, Harada K, Shigaki H, Miyake K, Kiyozumi Y, Ohuchi M, Kurashige J, Ishimoto T, Iwatsuki M, Sakamoto Y, Yoshida N (2016). UHRF1 regulates global DNA hypomethylation and is associated with poor prognosis in esophageal squamous cell carcinoma. Oncotarget.

[45] Ye J, Zhang Y, Liang W, Huang J, Wang L, Zhong X (2017). UHRF1 is an Independent Prognostic Factor and a Potential Therapeutic Target of Esophageal Squamous Cell Carcinoma. J Cancer.

[46] Ge M, Gui Z, Wang X, Yan F (2015). Analysis of the UHRF1 expression in serum and tissue for gastric cancer detection. Biomarkers.

[47] Mudbhary R, Hoshida Y, Chernyavskaya Y, Jacob V, Villanueva A, Fiel MI, Chen X, Kojima K, Thung S, Bronson RT, Lachenmayer A, Revill K, Alsinet C (2014). UHRF1 overexpression drives DNA hypomethylation and hepatocellular carcinoma. Cancer Cell.

[48] Liu X, Ou H, Xiang L, Li X, Huang Y, Yang D (2017). Elevated UHRF1 expression contributes to poor prognosis by promoting cell proliferation and metastasis in hepatocellular carcinoma. Oncotarget.

[49] Kim JH, Shim JW, Eum DY, Kim SD, Choi SH, Yang K, Heo K, Park MT (2017). Downregulation of UHRF1 increases tumor malignancy by activating the CXCR4/AKT-JNK/IL-6/Snail signaling axis in hepatocellular carcinoma cells. Sci Rep.

[50] Liang D, Xue H, Yu Y, Lv F, You W, Zhang B (2015). Elevated expression of UHRF1 predicts unfavorable prognosis for patients with hepatocellular carcinoma. Int J Clin Exp Pathol.

[51] Pi JT, Lin Y, Quan Q, Chen LL, Jiang LZ, Chi W, Chen HY (2013). Overexpression of UHRF1 is significantly associated with poor prognosis in laryngeal squamous cell carcinoma. Med Oncol.

[52] Li XT (2016). Identification of key genes for laryngeal squamous cell carcinoma using weighted co-expression network analysis. Oncol Lett.

[53] Zhao J, Chen X, Song G, Zhang J, Liu H, Liu X (2017). Uhrf1 controls the self-renewal versus differentiation of hematopoietic stem cells by epigenetically regulating the cell-division modes. Proc Natl Acad Sci U S A.

[54] Tan BT, Park CY, Ailles LE, Weissman IL (2006). The cancer stem cell hypothesis: a work in progress. Lab Invest.

[55] Huang P, Cheng CL, Chang YH, Liu CH, Hsu YC, Chen JS, Chang GC, Ho BC, Su KY, Chen HY, Yu SL (2016). Molecular gene signature and prognosis of non-small cell lung cancer. Oncotarget.

[56] Shi C, Zhang Z (2017). Screening of potentially crucial genes and regulatory factors involved in epithelial ovarian cancer using microarray analysis. Oncol Lett.

[57] Yan F, Wang X, Shao L, Ge M, Hu X (2015). Analysis of UHRF1 expression in human ovarian cancer tissues and its regulation in cancer cell growth. Tumour Biol.

[58] Cui L, Chen J, Zhang Q, Wang X, Qu J, Zhang J, Dang S (2015). Up-regulation of UHRF1 by oncogenic Ras promoted the growth, migration, and metastasis of pancreatic cancer cells. Mol Cell Biochem.

[59] Jeanblanc M, Mousli M, Hopfner R, Bathami K, Martinet N, Abbady AQ, Siffert JC, Mathieu E, Muller CD, Bronner C (2005). The retinoblastoma gene and its product are targeted by ICBP90: a key mechanism in the G1/S transition during the cell cycle. Oncogene.

[60] Benavente CA, Finkelstein D, Johnson DA, Marine JC, Ashery-Padan R, Dyer MA (2014). Chromatin remodelers HELLS and UHRF1 mediate the epigenetic deregulation of genes that drive retinoblastoma tumor progression. Oncotarget.

[61] Berdasco M, Gomez A, Rubio MJ, Catala-Mora J, Zanon-Moreno V, Lopez M, Hernandez C, Yoshida S, Nakama T, Ishikawa K, Ishibashi T, Boubekeur AM, Louhibi L (2017). DNA Methylomes Reveal Biological Networks Involved in Human Eye Development, Functions and Associated Disorders. Sci Rep.

[62] Rothbart SB, Krajewski K, Nady N, Tempel W, Xue S, Badeaux AI, Barsyte-Lovejoy D, Martinez JY, Bedford MT, Fuchs SM, Arrowsmith CH, Strahl BD (2012). Association of UHRF1 with methylated H3K9 directs the maintenance of DNA methylation. Nat Struct Mol Biol.

[63] Rajakumara E, Wang Z, Ma H, Hu L, Chen H, Lin Y, Guo R, Wu F, Li H, Lan F, Shi YG, Xu Y, Patel DJ (2011). PHD finger recognition of unmodified histone H3R2 links UHRF1 to regulation of euchromatic gene expression. Mol Cell.

[64] Xie S, Jakoncic J, Qian C (2012). UHRF1 double tudor domain and the adjacent PHD finger act together to recognize K9me3-containing histone H3 tail. J Mol Biol.

[65] Fang J, Cheng J, Wang J, Zhang Q, Liu M, Gong R, Wang P, Zhang X, Feng Y, Lan W, Gong Z, Tang C, Wong J (2016). Hemi-methylated DNA opens a closed conformation of UHRF1 to facilitate its histone recognition. Nat Commun.

[66] Tauber M, Fischle W (2015). Conserved linker regions and their regulation determine multiple chromatin-binding modes of UHRF1. Nucleus.

[67] Senft D, Qi J, Ronai ZA (2018). Ubiquitin ligases in oncogenic transformation and cancer therapy. Nat Rev Cancer.

[68] Jenkins Y, Markovtsov V, Lang W, Sharma P, Pearsall D, Warner J, Franci C, Huang B, Huang J, Yam GC, Vistan JP, Pali E, Vialard J (2005). Critical role of the ubiquitin ligase activity of UHRF1, a nuclear RING finger protein, in tumor cell growth. Mol Biol Cell.

[69] Guan D, Factor D, Liu Y, Wang Z, Kao HY (2013). The epigenetic regulator UHRF1 promotes ubiquitination-mediated degradation of the tumor-suppressor protein promyelocytic leukemia protein. Oncogene.

[70] Ma H, Chen H, Guo X, Wang Z, Sowa ME, Zheng L, Hu S, Zeng P, Guo R, Diao J, Lan F, Harper JW, Shi YG (2012). M phase phosphorylation of the epigenetic regulator UHRF1 regulates its physical association with the deubiquitylase USP7 and stability. Proc Natl Acad Sci U S A.

[71] De Vos M, El Ramy R, Quenet D, Wolf P, Spada F, Magroun N, Babbio F, Schreiber V, Leonhardt H, Bonapace IM, Dantzer F (2014). Poly(ADP-ribose) polymerase 1 (PARP1) associates with E3 ubiquitin-protein ligase UHRF1 and modulates UHRF1 biological functions. J Biol Chem.

[72] Gelato KA, Tauber M, Ong MS, Winter S, Hiragami-Hamada K, Sindlinger J, Lemak A, Bultsma Y, Houliston S, Schwarzer D, Divecha N, Arrowsmith CH, Fischle W (2014). Accessibility of different histone H3-binding domains of UHRF1 is allosterically regulated by phosphatidylinositol 5-phosphate. Mol Cell.

[73] Liu Y, Zhang B, Meng X, Korn MJ, Parent JM, Lu LY, Yu X (2017). UHRF2 regulates local 5-methylcytosine and suppresses spontaneous seizures. Epigenetics.

[74] Chen R, Zhang Q, Duan X, York P, Chen GD, Yin P, Zhu H, Xu M, Chen P, Wu Q, Li D, Samarut J, Xu G (2017). The 5-Hydroxymethylcytosine (5hmC) Reader UHRF2 Is Required for Normal Levels of 5hmC in Mouse Adult Brain and Spatial Learning and Memory. J Biol Chem.

[75] Lu H, Bhoopatiraju S, Wang H, Schmitz NP, Wang X, Freeman MJ, Forster CL, Verneris MR, Linden MA, Hallstrom TC (2016). Loss of UHRF2 expression is associated with human neoplasia, promoter hypermethylation, decreased 5-hydroxymethylcytosine, and high proliferative activity. Oncotarget.

[76] Zhou T, Xiong J, Wang M, Yang N, Wong J, Zhu B, Xu RM (2014). Structural basis for hydroxymethylcytosine recognition by the SRA domain of UHRF2. Mol Cell.

[77] Frauer C, Hoffmann T, Bultmann S, Casa V, Cardoso MC, Antes I, Leonhardt H (2011). Recognition of 5-hydroxymethylcytosine by the Uhrf1 SRA domain. PLoS One.

[78] Arita K, Ariyoshi M, Tochio H, Nakamura Y, Shirakawa M (2008). Recognition of hemi-methylated DNA by the SRA protein UHRF1 by a base-flipping mechanism. Nature.

[79] Avvakumov GV, Walker JR, Xue S, Li Y, Duan S, Bronner C, Arrowsmith CH, Dhe-Paganon S (2008). Structural basis for recognition of hemi-methylated DNA by the SRA domain of human UHRF1. Nature.

[80] Qian C, Li S, Jakoncic J, Zeng L, Walsh MJ, Zhou MM (2008). Structure and hemimethylated CpG binding of the SRA domain from human UHRF1. J Biol Chem.

[81] Bianchi C, Zangi R (2013). UHRF1 discriminates against binding to fully-methylated CpG-Sites by steric repulsion. Biophys Chem.

[82] Bronner C, Krifa M, Mousli M (2013). Increasing role of UHRF1 in the reading and inheritance of the epigenetic code as well as in tumorogenesis. Biochem Pharmacol.

[83] Berkyurek AC, Suetake I, Arita K, Takeshita K, Nakagawa A, Shirakawa M, Tajima S (2014). The DNA methyltransferase Dnmt1 directly interacts with the SET and RING finger-associated (SRA) domain of the multifunctional protein Uhrf1 to facilitate accession of the catalytic center to hemi-methylated DNA. J Biol Chem.

[84] Bashtrykov P, Jankevicius G, Jurkowska RZ, Ragozin S, Jeltsch A (2014). The UHRF1 protein stimulates the activity and specificity of the maintenance DNA methyltransferase DNMT1 by an allosteric mechanism. J Biol Chem.

[85] Liang CC, Zhan B, Yoshikawa Y, Haas W, Gygi SP, Cohn MA (2015). UHRF1 is a sensor for DNA interstrand crosslinks and recruits FANCD2 to initiate the Fanconi anemia pathway. Cell Rep.

[86] Greiner VJ, Kovalenko L, Humbert N, Richert L, Birck C, Ruff M, Zaporozhets OA, Dhe-Paganon S, Bronner C, Mely Y (2015). Site-Selective Monitoring of the Interaction of the SRA Domain of UHRF1 with Target DNA Sequences Labeled with 2-Aminopurine. Biochemistry.

[87] Myrianthopoulos V, Cartron PF, Liutkeviciute Z, Klimasauskas S, Matulis D, Bronner C, Martinet N, Mikros E (2016). Tandem virtual screening targeting the SRA domain of UHRF1 identifies a novel chemical tool modulating DNA methylation. Eur J Med Chem.

[88] Das PM, Singal R (2004). DNA methylation and cancer. J Clin Oncol.

[89] Yu C, Xing F, Tang Z, Bronner C, Lu X, Di J, Zeng S, Liu J (2013). Anisomycin suppresses Jurkat T cell growth by the cell cycle-regulating proteins. Pharmacol Rep.

[90] Abusnina A, Keravis T, Yougbare I, Bronner C, Lugnier C (2011). Anti-proliferative effect of curcumin on melanoma cells is mediated by PDE1A inhibition that regulates the epigenetic integrator UHRF1. Mol Nutr Food Res.

[91] Du S, Xu G, Zou W, Xiang T, Luo Z (2017). Effect of dihydroartemisinin on UHRF1 gene expression in human prostate cancer PC-3 cells. Anticancer Drugs.

[92] Boukhari A, Alhosin M, Bronner C, Sagini K, Truchot C, Sick E, Schini-Kerth VB, Andre P, Mely Y, Mousli M, Gies JP (2015). CD47 activation-induced UHRF1 over-expression is associated with silencing of tumor suppressor gene p16INK4A in glioblastoma cells. Anticancer Res.

[93] Lin Y, Chen W, Wang Z, Cai P (2017). Emodin promotes the arrest of human lymphoma Raji cell proliferation through the UHRF1DNMT3ANp73 pathways. Mol Med Rep.

[94] Santhekadur PK, Rajasekaran D, Siddiq A, Gredler R, Chen D, Schaus SE, Hansen U, Fisher PB, Sarkar D (2012). The transcription factor LSF: a novel oncogene for hepatocellular carcinoma. Am J Cancer Res.

[95] Grant TJ, Bishop JA, Christadore LM, Barot G, Chin HG, Woodson S, Kavouris J, Siddiq A, Gredler R, Shen XN, Sherman J, Meehan T, Fitzgerald K (2012). Antiproliferative small-molecule inhibitors of transcription factor LSF reveal oncogene addiction to LSF in hepatocellular carcinoma. Proc Natl Acad Sci U S A.

[96] Chin HG, Ponnaluri VK, Zhang G, Esteve PO, Schaus SE, Hansen U, Pradhan S (2016). Transcription factor LSF-DNMT1 complex dissociation by FQI1 leads to aberrant DNA methylation and gene expression. Oncotarget.

[97] Leon-Gonzalez AJ, Jara-Palacios MJ, Abbas M, Heredia FJ, Schini-Kerth VB (2017). Role of epigenetic regulation on the induction of apoptosis in Jurkat leukemia cells by white grape pomace rich in phenolic compounds. Food Funct.

[98] Seo JS, Choi YH, Moon JW, Kim HS, Park SH (2017). Hinokitiol induces DNA demethylation via DNMT1 and UHRF1 inhibition in colon cancer cells. BMC Cell Biol.

[99] Krifa M, Alhosin M, Muller CD, Gies JP, Chekir-Ghedira L, Ghedira K, Mely Y, Bronner C, Mousli M (2013). Limoniastrum guyonianum aqueous gall extract induces apoptosis in human cervical cancer cells involving p16 INK4A re-expression related to UHRF1 and DNMT1 down-regulation. J Exp Clin Cancer Res.

[100] Kim MY, Park SJ, Shim JW, Yang K, Kang HS, Heo K (2015). Naphthazarin enhances ionizing radiation-induced cell cycle arrest and apoptosis in human breast cancer cells. Int J Oncol.

[101] Wang C, Wang X, Su Z, Fei H, Liu X, Pan Q (2015). The novel mTOR inhibitor Torin-2 induces autophagy and downregulates the expression of UHRF1 to suppress hepatocarcinoma cell growth. Oncol Rep.

[102] Jang SY, Hong D, Jeong SY, Kim JH (2015). Shikonin causes apoptosis by up-regulating p73 and down-regulating ICBP90 in human cancer cells. Biochem Biophys Res Commun.

[103] Huang G, Zhao HR, Meng QQ, Zhang QJ, Dong JY, Zhu BQ, Li SS (2018). Synthesis and biological evaluation of sulfur-containing shikonin oxime derivatives as potential antineoplastic agents. Eur J Med Chem.

[104] Houliston RS, Lemak A, Iqbal A, Ivanochko D, Duan S, Kaustov L, Ong MS, Fan L, Senisterra G, Brown PJ, Wang YX, Arrowsmith CH (2017). Conformational dynamics of the TTD-PHD histone reader module of UHRF1 reveals multiple histone binding states, allosteric regulation and druggability. J Biol Chem.

[105] Kwon K, Kwon YS, Kim SW, Yu K, Lee KH, Kwon OY (2017). Luteolin-induced apoptosis through activation of endoplasmic reticulum stress sensors in pheochromocytoma cells. Mol Med Rep.

[106] Chen P, Zhang JY, Sha BB, Ma YE, Hu T, Ma YC, Sun H, Shi JX, Dong ZM, Li P (2017). Luteolin inhibits cell proliferation and induces cell apoptosis via down-regulation of mitochondrial membrane potential in esophageal carcinoma cells EC1 and KYSE450. Oncotarget.

[107] Ding S, Hu A, Hu Y, Ma J, Weng P, Dai J (2014). Anti-hepatoma cells function of luteolin through inducing apoptosis and cell cycle arrest. Tumour Biol.

[108] Liu Y, Lang T, Jin B, Chen F, Zhang Y, Beuerman RW, Zhou L, Zhang Z (2017). Luteolin inhibits colorectal cancer cell epithelial-to-mesenchymal transition by suppressing CREB1 expression revealed by comparative proteomics study. J Proteomics.

[109] Deng L, Jiang L, Lin X, Tseng KF, Lu Z, Wang X (2017). Luteolin, a novel p90 ribosomal S6 kinase inhibitor, suppresses proliferation and migration in leukemia cells. Oncol Lett.

[110] Lin TH, Hsu WH, Tsai PH, Huang YT, Lin CW, Chen KC, Tsai IH, Kandaswami CC, Huang CJ, Chang GD, Lee MT, Cheng CH (2017). Dietary flavonoids, luteolin and quercetin, inhibit invasion of cervical cancer by reduction of UBE2S through epithelial-mesenchymal transition signaling. Food Funct.

[111] Song S, Su Z, Xu H, Niu M, Chen X, Min H, Zhang B, Sun G, Xie S, Wang H, Gao Q (2017). Luteolin selectively kills STAT3 highly activated gastric cancer cells through enhancing the binding of STAT3 to SHP-1. Cell Death Dis.

[112] Manigandan K, Manimaran D, Jayaraj RL, Elangovan N, Dhivya V, Kaphle A (2015). Taxifolin curbs NF-kappaB-mediated Wnt/beta-catenin signaling via up-regulating Nrf2 pathway in experimental colon carcinogenesis. Biochimie.

[113] Hossain MM, Ray SK (2014). EWS Knockdown and Taxifolin Treatment Induced Differentiation and Removed DNA Methylation from p53 Promoter to Promote Expression of Puma and Noxa for Apoptosis in Ewing's Sarcoma. J Cancer Ther.

[114] Davatgaran-Taghipour Y, Masoomzadeh S, Farzaei MH, Bahramsoltani R, Karimi-Soureh Z, Rahimi R, Abdollahi M (2017). Polyphenol nanoformulations for cancer therapy: experimental evidence and clinical perspective. Int J Nanomedicine.

[115] Yang CJ, Wang ZB, Mi YY, Gao MJ, Lv JN, Meng YH, Yang BY, Kuang HX (2016). UHPLC-MS/MS Determination, Pharmacokinetic, and Bioavailability Study of Taxifolin in Rat Plasma after Oral Administration of its Nanodispersion. Molecules.

[116] Zu Y, Wu W, Zhao X, Li Y, Wang W, Zhong C, Zhang Y, Zhao X (2014). Enhancement of solubility, antioxidant ability and bioavailability of taxifolin nanoparticles by liquid antisolvent precipitation technique. Int J Pharm.

